# The Institutional Worker

**Published:** 1912-12-21

**Authors:** 


THE
### INSTITUTIONAL WORKER
Being a Special Supplement to "The Hospital"

### BUREAU OF INFORMATION.

### Rules for Correspondents.

1. Every letter, on whatever subject, must be accompanied by the coupon to be cut from the back cover (inside page) of The Hospital, current issue, and must contain the name and address of the correspondent with pseudonym for publication if desired.

2. Replies by post cannot be given, save under exceptional circumstances at the Editor's discretion.

3. Letters from Approved Homes in reply to special needs published in the Bureau should state terms and full particulars, and be sent prepaid under cover to the Editor of the Bureau with name written across coupon for identification.

4. Proprietors of Homes which have not yet been entered on the List of Approved Homes, but have spare accommodation likely to suit special needs, are invited to write for an application form for registration. The fee for registration, which includes two announcements of the Home in the Bureau, is 5s.

5. Special needs of every description relating to those who are sick or in difficulties are made known in these columns free of charge.

6. All communications to be addressed to The Editor of The Hospital, 28 Southampton Street, Strand, London, W.C., and marked "Bureau of Information."

### Approved Homes.

Note.—*No Home is added to the List without direct medical and other testimony to its good management.*

**Woodford Green.** — Forest Hall, Hale End, near Epping Forest. Principals: Miss Garrard and Miss von Limburg. Delicate children and (in separate wing) a few mentally deficient and paralysed. Large garden. Education and skilled nursing.

**South Devon.**—West Brimley House, Teignmouth. Principals: Miss Gladwin and Miss Hewlett. Fully equipped Nursing Home for medical, surgical, maternity, and other cases, not mental. Massage. Facilities for open-air treatment. Mild climate. Special care given to delicate children.

### Needs and Helpers.

**Home for Cancer Patient.**—The patient is a lady, aged sixty-one, suffering from cancer in the breast, with symptoms which threaten rapid development. Apply to the Sister-in-Charge, The Hostel of God, 29 North Side, Clapham Common, S.W. The Home is quite free and under admirable management. We have forwarded the name of a Home where she could perhaps be received at 10s. 6d. a week.—E. C. S.

**Free Convalescent Aid.** — The patient is a young woman suffering from excessive weakness, and needs care and good food. Write yourself to Mrs. Kitto, Free Convalescent Home, South Park, Reigate, and state the age and circumstances of your patient more fully, and the cause of her present condition.—Nurse Alice C.

**Convalescent Aid for Nurse.**—You can hardly do better than send your nurse who is suffering from nervous debility to the Merchant Taylors' Company's Convalescent Home for Ladies at Bognor. Write to the Clerk, The Hall, Threadneedle Street, E.C. Free railway pass is given from London, and there is no charge. If this is too far, write to the Matron, the Frederica Countess of Scarbrough's Convalescent Home, Skegness; payment 7s. 6d. a week. It re-opens in January.—Dr. H., Gateshead.

### Training and Employment.

**Massage Training for Nurse.**—Write to the Matron of one of the following hospitals: The National Hospital, Queen Square, Bloomsbury; the West End Hospital, Welbeck Street, W.; the Hospital for Epilepsy and Paralysis, Maida Vale, W., and get particulars of their school of massage and electricity. State your qualifications and ask whether services could be accepted in lieu of payment. As an old "King's" nurse you have a good chance.—Massage.

**Employment for Deserted Wife.** — The young woman in question will not succeed in obtaining more than bare maintenance if she makes it a condition to retain with her her boy aged three and a-half. Caretakers are usually selected by local recommendation, not from advertisements, and as they are expected to sleep alone in the house, older women would be preferred. We strongly recommend your case to go into service and place her boy with a reliable person near at hand.—Miss Edith E. G. C., Faringdon.

**Home Work for Retired Nurse.**—It is a difficult thing to get the care of a child who could pay enough to help. Your best chance would be with defective children, for there is a considerable demand for accommodation for these at reasonable terms. With your special training and experience in this branch of work you might succeed well. Let us know if you would like a form for registration.—E. C.

### Nursing in the Empire and Abroad.

**Conditions in the Fiji Islands.**—Medical matters are distinctly advanced in these islands, and at Suva, the principal town, a Nursing Home, principally for maternity patients, will be opened early in the year. The Colonial Hospital there trains its own nurses and maintains a high standard by getting them examined and certified through the Australasian Nurses' Association. But there are often occasions when the supply of nurses is not equal to the demand, and a British nurse going out as you wish to do for a year would probably meet with a kind reception, much hospitality, and a good share of work. We recommend you to write to the Chief Medical Officer, Suva, Fiji Islands.—R. O.

**Anti-English Feeling in Greece.**—We are very happy to have your letter, which appears in another part of our columns, and to know that the feeling against English nurses reported to us a short time back on high official authority was merely temporary, and has now entirely disappeared. Greece has for generations commanded the sympathy of Great Britain, and it is good to learn Englishwomen are playing their part in helping to assuage suffering in this crisis.—Mme. Mariette Kephale.

**Zammitt Clapp Hospital, Malta.**—Your beautiful photographs are much appreciated, and we have pleasure in forwarding to you the copies of The Hospital you require.—Dr. Alacogne.

### Miscellaneous.

**Partner for Nursing Home.**—We can only recommend you either to advertise for some weeks continuously or to consult a good agent, who could no doubt put you in communication with ladies desirous of meeting with the opportunity you offer. With regard to the sale of spare theatre requisites, you will notice we have lately opened a "Sale and Exchange" Column which exactly meets your need.—I. M. P.

### Letter-Box.

**The Editor begs to acknowledge the receipt of communications, with enclosures, from:**—Mrs N. H., Bexhill; Miss D., Staines; Miss F., Worthing; Miss M. von L., Forest Hill; the Misses E., Dover; the Misses G. and H., Teignmouth.

**Communications have been received and attended to from:**—Miss E., Dover; Miss E. T. S., Blackheath.

**The Editor is much indebted for testimonials in response to inquiries from:**—Mrs. Nash, Mrs. Hill, and Dr. Chappel.

We have forwarded your letter to the Business Manager. We quite sympathise with you in the low terms suggested. —L. S.

We are endeavouring to procure for you the information you require, and will answer your query fully next week.—H. B.

Many thanks. We will not fail to forward you a list of our Approved Nursing Homes early in the New Year.—SECRETARY, INVALID CHILDREN'S AID ASSOCIATION.

Many thanks for information received:—NURSE-SUPERINTENDENT, BRITISH GUIANA; K. M. L.; DISTRESSED WIDOWS' SOCIETY.

We are sending on to you particulars of the demand for nurses at Suez and trust the information will serve to secure them what they need.—PLYMOUTH; ANGLAISE.

**Persons requiring nurses and nurses requiring appointments will find their needs met in the adjoining pages, where many useful announcements appear this week.**

### Housekeeping Queries.

**Mock Venison.**—No wonder the staff are tired of so much mutton. Try cooking it as follows: Take a shoulder or leg of mutton and hang for a week; then put it into a basin large enough to let it lie flat, with half a pint of vinegar and the same quantity of water; take $\frac{1}{4}$ oz. each of pounded allspice and black pepper and rub the mutton with this. The joint should lie in the liquid for about a week and be turned every day. Cook in the same manner as venison and serve with thick gravy and currant jelly. If there are any pieces of the fat end of bacon in the larder this can be utilised for larding.—DISSATISFIED.

**Waste of Matches.**—We are always delighted to give any hints which may promote economy. You will find it quite possible to check the number of boxes of matches used even in your large establishment if you will have them given out on a fixed day in the week by the housekeeper to each worker individually. Experience will soon show how long a box should last, and the requirements of the more dependable among the staff must regulate that of the others. Each one should have a little locker in which to keep her own stores, such as house flannel, soap, matches, etc., and should know exactly how long her allowance is to last. We have known young housemaids run about with a dozen boxes of matches concealed in their aprons when no supervision was exercised and access was had to a common store. One more hint. Do not disdain to ascertain whether your young maids know how to light a match. If you watch you will often see them striking three or four before they get a light, and when this is going on every day in every part of the house dozens of boxes are wasted without anyone knowing why.—PUZZLED MATRON.

### Institution Facts and Figures.

### AN INVITATION TO THE PRACTICAL WORKER.

IN every well-managed institution there is an accumulation of valuable observations, extending often over a term of years, which are wasted because there is no medium through which they can be brought to the notice of fellow-workers in the same field. It is our hope through this section of the "Institutional Worker" to collect some of these facts and present them to our readers. Every month two questions will be set relating to the internal working of institutional life, its cost, and distinctive features. The best answers will be published in due course, and payment will be made for contributions at the rate of Five Shillings for each answer which the Editor considers of sufficient interest for publication.

QUESTIONS FOR DECEMBER.

1. What was the exact amount of milk consumed in a given week in December in your institution (*a*) in a medical ward; (*b*) in a surgical ward? State daily average of patients. Describe the system in use for checking the amount used. What was the average cost per head of each patient in those wards respectively for milk during that week calculated on the contract price?

2. What is the most popular supper dish (savoury) in your institution? Give the recipe and quantity of each ingredient required for fifty persons, and state the cost per head.

The figures published by King Edward's Hospital Fund relating to the cost of various articles in London hospitals have brought forcibly before the public the variations which occur in the cost of everyday articles in London institutions. If our readers will help us we hope to throw additional light on the hospital commissariat throughout the kingdom.

Answers are invited from workers in every class of institution for the sick, and the value of those published will be greatly enhanced if opportunity be afforded for contrasting figures in various parts of the kingdom. The name of the institution need not be mentioned unless desired. The only condition is that the figures must be the result of actual observation and computation, not estimates. Either or both of the questions may be answered by each contributor, and payment will be made for all answers published as above mentioned.

The following rules must be observed by contributors to the FACTS AND FIGURES Column:—

1. Answers must be written on one side of the paper and must bear the name and address of the sender and be accompanied by coupon to be cut from the back cover (inside page) of the current issue of THE HOSPITAL. A pseudonym must be chosen if the name is not to be published.

2. Answers must be addressed to the Editor of THE HOSPITAL, 28-29 Southampton Street, Strand, London, W.C.; they must be marked in the left-hand corner "Facts and Figures," and must be received before the last day of the current month.

### Editor's Notices.

**Contributions:**

Contributions should be written, or preferably typed, on one side of the paper only, and all articles sent in are accepted upon the distinct understanding that they are forwarded to THE HOSPITAL only.

The Editor cannot undertake to return MSS. not used, but when a stamped directed envelope is enclosed the MSS. may be returned if a special request is made.

Accepted articles and paragraphs of news will be paid for after publication at the scale rate.

**Address:**

To prevent delay all contributions and letters on editorial business must be addressed exclusively to the Editor, "The Hospital," 29 Southampton Street, Strand, London, W.C. It is important that this regulation shall be strictly observed.

**Correspondence:**

Correspondence on all subjects is invited. The name and address of correspondents must be given as a guarantee of good faith, but not necessarily for publication.

**Special Articles:**

Special articles are invited, and questions, inquiries, and paragraphs upon all matters relating to the work, administration, and management of general, special, mental, fever, cottage and convalescent hospitals, sanatoria, homes, institutions, societies and organisations for the treatment or care of the sick, injured and dependants of all classes. Every matter affecting the interests and welfare of the staff of all grades working in these institutions will receive special consideration.

**Administrative Medicine, Research and Items of News:**

Special terms will be paid for approved articles on subjects in this wide field by experts who have made some section of it their special study and interest. We wish to give prominence to every movement tending to advance accuracy of diagnosis, efficiency in treatment, and the development of modern methods to eradicate disease and promote the welfare of the suffering.

**A Bureau of Information:**

We invite inquiries and applications for information and help in providing for that numerous class of sufferers who require special care or house-room which they cannot obtain in their own homes or secure for themselves. **We cannot, however, prescribe, or recommend practitioners.**

**Books for Review:**

Publishers are particularly requested to send advance proofs of any new books of importance whenever possible, as the Editor has made arrangements to publish immediate reviews on a new plan.

**Photographs, Plans, Blocks, and Illustrations:**

It is requested that wherever possible MSS. may be accompanied by illustrations in any of the above forms. The name of the sender and of the article to which it belongs should be written on the back of each photograph, drawing, or block for purposes of identification.

**Local Papers:**

Newspapers containing reports or news paragraphs should be marked and addressed to the Sub-Editor.

**The Coupon System:**

A coupon will be found at the bottom of the third inside page of the cover each week. This coupon must be attached to every question or inquiry to which an answer is desired in THE HOSPITAL.

### Start the New Year Well.

THERE is no Journal other than THE HOSPITAL that so comprehensively covers the whole Institutional World. It is essential not only to those responsible for the Construction, Administration, and Maintenance of Institutions generally, but to every individual worker in any department of the Medical, Health, and Sanitary Services. Useful and authoritative articles on Institution, Poor-Law, and Municipal Affairs, as well as the all-important question of National Insurance, are contained within its columns. To keep abreast with the progress in all branches of Institutional Work, it is necessary to secure a copy of THE HOSPITAL every week; to miss even one Number is an irreparable loss, which no keen worker can afford. In these circumstances, to start the New Year well you should place a permanent order with your Newsagents, so as to ensure that you obtain a copy of THE HOSPITAL every Thursday, or a subscription may be forwarded to the Publishers at the undermentioned rates.

**Subscriptions,** which may commence at any time, are payable in advance. Cheques and Post-Office Orders should be crossed London County and Westminster Bank, Covent Garden Branch, and made payable to the Manager of THE HOSPITAL.

RATES OF SUBSCRIPTION (payable in advance).

| | | |
|---|---|---|
| **UNITED KINGDOM.** | | |
| Three Months (including Postage) | ... ... ... ... | 2s. 0d. |
| Six Months do. | ... ... ... ... | 4s. 0d. |
| Twelve Months do. | ... ... ... ... | 6s. 6d. |
| **FOREIGN AND COLONIAL.** | | |
| Three Months (including Postage) | ... ... ... ... | 2s. 6d. |
| Six Months do. | ... ... ... ... | 5s. 0d. |
| Twelve Months do. | ... ... ... ... | 8s. 8d. |

*SUBSCRIPTION FORM.*

*Please send me* THE HOSPITAL *for..............months, commencing with your issue of.............................., for which please find enclosed..............................*

*Name*..................................................

*Address* ..................................................

..................................................

*Date*....................................

*To the Manager,*
*"The Hospital" Building,*
*28 & 29 Southampton Street,*
*Strand, London, W.C.*

### Manager's Notices.

**All letters on business matters must be addressed to The Manager, "The Hospital," 28 and 29 Southampton Street, London, W.C.**

**Advertisements:**

To ensure insertion, all advertisements for THE HOSPITAL must reach the Manager not later than Wednesday morning in each week. For scale of charges see pages v and 4.

**Special Rates** will be quoted for those institutions that agree to send all their vacancy, contract, and public notices to THE HOSPITAL each year, so as to make it a file of such announcements for ready reference.

**Remittances:**

**It is especially requested that remittances be made by Cheque or Postal Order, and in halfpenny stamps only when the amount is under sixpence.**

